# Multicenter retrospective cohort study demonstrates superior safety profile of indobufen over aspirin for Post-CABG antiplatelet therapy

**DOI:** 10.3389/fphar.2024.1474150

**Published:** 2024-09-30

**Authors:** Yu Ren, Yanwu Zhu, Qiaoyan Yan, Hui Jin, Hua Luo

**Affiliations:** ^1^ Department of Pharmacy, Taizhou Hospital of Zhejiang Province Affiliated to Wenzhou Medical University, Taizhou, Zhejiang, China; ^2^ Department of Orthopedic, Taizhou Hospital of Zhejiang Province Affiliated to Wenzhou Medical University, Taizhou, Zhejiang, China

**Keywords:** aspirin, indobufen, coronary artery bypass grafting, myocardial infarction, complication

## Abstract

**Objectives:**

Coronary artery bypass grafting (CABG) is essential for treating coronary artery disease, with postoperative aspirin crucial to prevent graft restenosis. However, its gastrointestinal side effects may limit tolerability in some patients. Indobufen presents a potential alternative, but its safety and efficacy need further validation. This study aimed to compare the efficacy and safety of indobufen *versus* aspirin in patients’ post-CABG.

**Methods:**

This retrospective observational study included 39 patients who underwent CABG at two centers from January to December 2023. Patients were retrospectively assigned to two groups based on the antiplatelet therapy they received: the indobufen group (n = 19) and the aspirin group (n = 20). The primary endpoint was a composite of non-fatal myocardial infarction, stroke, and revascularization due to acute coronary syndrome in the intention-to-treat population. Postoperative data on platelet count, hemoglobin, D-dimer, activated partial thromboplastin time (APTT), and hospital stay length were collected. Transfusion rate, bleeding, thrombotic events, and gastrointestinal adverse reactions were compared between the two groups.

**Results:**

Over the 8-to-18-month follow-up period, 5 patients (25%) in the aspirin group reached the primary endpoint, while none in the indobufen group did, a difference that was statistically significant (*p* = 0.02). Although the rates of non-fatal myocardial infarction, revascularization, stroke, and thrombotic events were higher in the aspirin group, these differences did not reach statistical significance. Importantly, the total bleeding events were markedly lower in the indobufen group (15.79% vs. 55%, *p* = 0.011), with major bleeding events also significantly reduced in the indobufen group (0% vs. 20%, *p* = 0.04). Both groups showed no significant differences were observed in postoperative hospital stay, hemoglobin, and D-dimer levels between the groups. However, the indobufen group demonstrated significantly lower platelet count and APTT. The average daily cost of indobufen was 27.8 times higher than that of aspirin.

**Conclusion:**

Indobufen demonstrates a comparable antiplatelet effect to aspirin and offers significant advantages in reducing gastrointestinal adverse reactions and bleeding risk. It can be considered a preferable alternative for patients who cannot tolerate or have contraindications to aspirin. Further large-scale clinical trials are needed to confirm its potential as the first-choice antiplatelet therapy post-CABG.

## Introduction

Coronary atherosclerosis can lead to severe consequences such as coronary artery stenosis, myocardial ischemia, and myocardial infarction. When unstable plaques rupture, platelets accumulate and adhere to the rupture site, forming fibrin and activating the coagulation system, which results in thrombosis (Wu et al., 2019). Coronary artery bypass grafting (CABG) is a highly effective treatment for coronary heart disease, as it establishes collateral circulation to improve myocardial ischemia and hypoxia (Doenst et al., 2019). However, restenosis of the graft vessel can occur post-CABG, leading to the failure of the re-established collateral circulation, recurring myocardial ischemia, and hypoxia. This increases the likelihood of secondary revascularization and long-term patient mortality (Zhao et al., 2018). Platelet activation and aggregation are crucial in the development of atherothrombosis, making antiplatelet therapy vital in treating coronary heart disease (Davì and Patrono, 2007; Gaudino et al., 2017).

Aspirin is a widely used antiplatelet drug that reduces the synthesis and release of thromboxane A2, inhibiting platelet aggregation and thrombosis (Johnston et al., 2019). However, long-term use of aspirin is associated with a high incidence of gastrointestinal injury and bleeding, leading to poor patient tolerance. Consequently, guidelines and textbooks recommend indobufen as an alternative for aspirin-intolerant patients who are at high risk of bleeding and gastric ulcers (Yong Huo et al., 2021; Specialty Committee on Prevention and Treatment of Thrombosis of Chinese College of Cardiovascular Physicians et al., 2019). While aspirin’s role in preventing and treating cardiovascular and cerebrovascular diseases is well-supported by substantial evidence, research evidence for indobufen remains relatively limited. This study compared the safety and efficacy of indobufen and aspirin following CABG, aiming to provide clinical references for the use of indobufen and to inform future large-scale multicenter prospective studies.

## Methods

### Study design and participants

This study was a retrospective study and the work has been reported in line with the STROCSS criteria (Mathew et al., 2021). Patients with coronary heart disease who underwent coronary artery bypass grafting in two centers from January 2023 to December 2023 were selected. Inclusion criteria: (Wu et al., 2019): Patients were diagnosed with coronary heart disease and underwent coronary artery bypass grafting; (Doenst et al., 2019); Saphenous vein and internal mammary artery were used as grafts; (Zhao et al., 2018); Patients without a history of allergy to aspirin, clopidogrel, and indobufen; (Davì and Patrono, 2007); Aspirin was discontinued 5–7 days before surgery. Exclusion criteria: (Wu et al., 2019): Complicated with serious organic diseases of other systems; (Doenst et al., 2019); Patients with coagulation dysfunction or bleeding tendency; (Zhao et al., 2018); Patients with malignant tumors; (Davì and Patrono, 2007); Atrial fibrillation (Gaudino et al., 2017) Patients undergoing other types of cardiac surgery (such as valve replacement) at the same time; (Johnston et al., 2019); Other patients requiring anticoagulant drugs. This study was approved by the Ethics Committee in our hospital (Approval No.: KL20240428).

### Group assignment and interventions

Participants were divided into two groups retrospectively based on the type of postoperative antiplatelet therapy they received: the indobufen group and the aspirin group. The aspirin group was selected based on a matching principle, ensuring similarity in age, gender, and underlying conditions with the indobufen group, to minimize potential confounding bias due to differences in patient characteristics. This study was conducted across two centers (Taizhou Hospital and Enze Hospital) to enhance the generalizability of the findings. Clinical data such as age, gender, course of disease, body mass index (BMI), medical history, coronary heart disease risk factors, and family history were collected through the electronic medical record system.

Patients in both groups underwent off-pump CABG and received coronary artery dilation, heart rate reduction, and blood lipid regulation after surgery. The aspirin group were treated with aspirin and clopidogrel (oral aspirin enteric-coated tablets 100 mg daily, clopidogrel bisulfate tablets 75 mg daily). The indobufen group was treated with indobufen and clopidogrel (oral indobufen tablets 100 mg twice daily, clopidogrel bisulfate tablets 75 mg daily).

### Data collection

Observation indicators included postoperative platelet count (PLT), hemoglobin (Hb), D-dimer, activated partial thromboplastin time (APTT) levels, length of hospital stay, transfusion rate and incidence of adverse reactions. Main adverse reactions included bleeding events and thrombotic events.

## Outcomes

The primary endpoint was a composite of all-cause death, non-fatal myocardial infarction, stroke, revascularization due to acute coronary syndrome, and major bleeding complications during the follow-up period (8–18months). Major bleeding was defined as Bleeding Academic Research Consortium (BARC) type 3 or higher bleeding. The individual components of the primary endpoint, revascularization, and minor gastrointestinal complications were analyzed as secondary endpoints at 18 months. Interim analysis was not planned or conducted during the follow-up duration. Post-hoc secondary composite endpoints included thrombotic composite endpoint (cardiac death, non-fatal myocardial infarction, ischemic stroke, revascularization, definite or probable stent thrombosis) and any bleeding (BARC type ≥2 bleeding).

### Statistical analysis

Categorical data are presented as absolute numbers and percentages (n, %), while continuous data are presented as means and standard deviation. SPSS 25.0 software was used for statistical analysis. Continuous data conforming to a normal distribution were described as mean ± SD, and comparisons between groups were conducted using two independent sample t-tests. Categorical data were analyzed using the chi-square test. A *p*-value of <0.05 was considered statistically significant.

## Results

**FIGURE 1:**
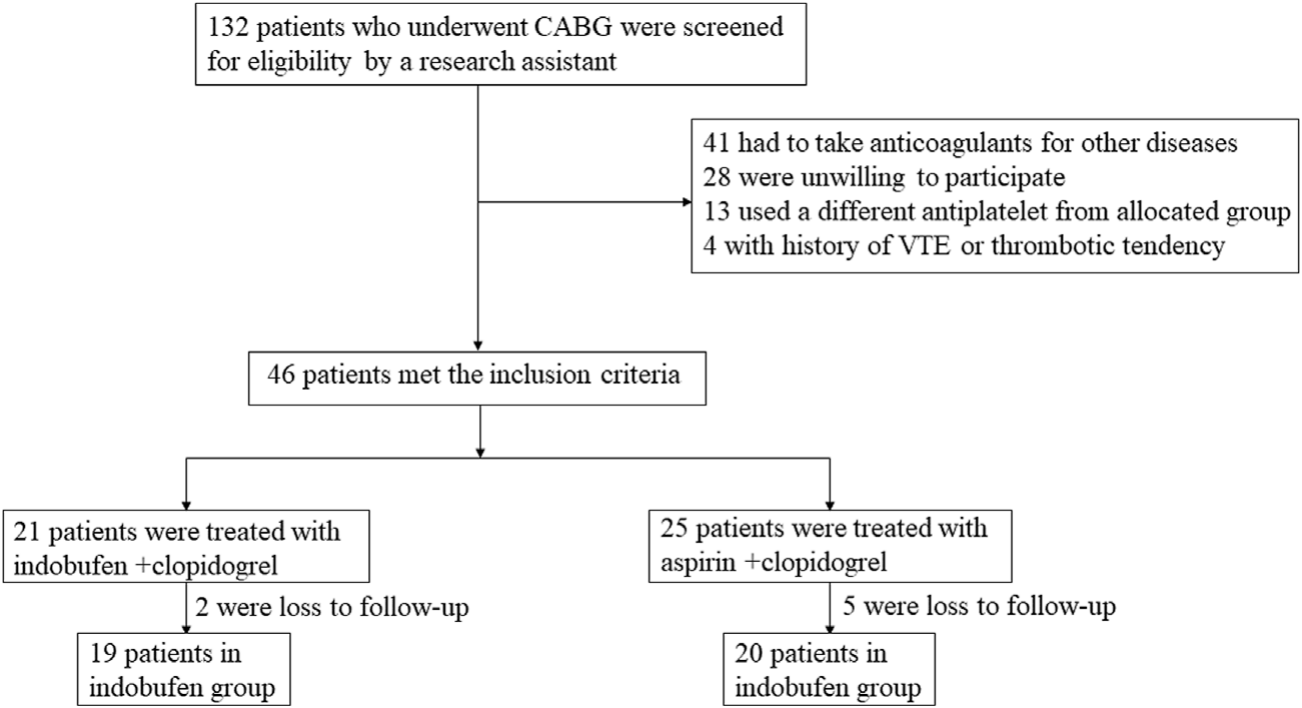
Selection process for study participants.

A total of 39 patients with coronary heart disease undergoing CABG were enrolled (the selection process is shown in Figure 1). The baseline characteristics of the total population are shown in Table 1. There were 19 patients in the indobufen group, including 4 females (21.05%), aged 63.84 ± 7.99 years. There were 20 patients in the aspirin group, including 4 females (20%), aged 64.60 ± 6.72 years. The two groups were well-balanced for demographic, clinical, and procedural characteristics and non-trial-related medications. There were no significant differences in gender, age, BMI, hypertension, diabetes, hyperlipidemia, history of cerebral infarction, history of myocardial infarction, and peripheral arterial plaque between the two groups (*p* > 0.05).

**TABLE 1:** The baseline characteristics of the total population.

Group	Indobufen (n = 19)	Aspirin (n = 20)
Age (Mean ± SD)	63.84 ± 7.99	64.60 ± 6.72
Sex (Female/male)	4/15	4/16
BMI (Mean ± SD)	23.43 ± 2.48	25.88 ± 3.17
Hypertension (%)	10 (52.63%)	13 (65%)
Diabetes (%)	9 (47.37%)	10 (50%)
Hyperlipidemia (%)	4 (21.05%)	3 (15%)
Ischemic stroke (%)	8 (42.11%)	4 (20%)
Myocardial infarction (%)	7 (36.84%)	4 (20%)
Peripheral arterial plaque (%)	16 (84.21%)	17 (85%)

BMI, body mass index.

During the 8–18 months follow-up, the primary endpoint occurred in 5 (25%) patients who received aspirin and in none who received indobufen (*p* = 0.02), indicating an absolute risk reduction. The incidences of the individual components of the primary endpoint and other secondary endpoints are shown in Table 2. During the 8–18 months follow-up, no patients died in either group. The incidence of non-fatal myocardial infarction, revascularization, stroke, and thrombotic events were lower in the indobufen group than in the aspirin group. Regarding the primary endpoint, the beneficial effect of indobufen compared with aspirin was generally consistent across all prespecified subgroups (Table 2).

**TABLE 2:** Major efficacy and safety endpoints during follow-up.

Group	Indobufen (n = 19)	Aspirin (n = 20)	*p*-value
**Primary efficacy endpoint (%)**	0 (0%)	5 (25%)	0.02
Non-fatal myocardial infarction (%)	0 (0%)	2 (10%)	0.157
Revascularization (%)	0 (0%)	1 (5%)	0.323
Stroke (%)	0 (0%)	1 (5%)	0.323
Thrombotic event (%)	0 (0%)	2 (10%)	0.157
**Secondary safety endpoint (%)**	3 (15.79%)	14 (70%)	0.01
Total bleeding events (%)	3 (15.79%)	11 (55%)	0.011
Minor bleeding events (%)	3 (15.79%)	7 (35%)	0.17
Major bleeding events (%)	0 (0%)	4 (20%)	0.04
Gastrointestinal reaction (%)	0 (0%)	3 (15%)	0.079
**Other prognostic indicators**			
Postoperative hospital (Mean ± SD)	13.95 ± 5.99	13.60 ± 5.91	0.887
PLT (Mean ± SD)	199.58 ± 87.16	303.70 ± 100.50	0.001
Hb (Mean ± SD)	103.84 ± 19.49	105.05 ± 17.95	0.841
D-dimer (Mean ± SD)	1.12 ± 0.71	1.23 ± 0.92	0.656
APTT (Mean ± SD)	39.79 ± 4.63	44.64 ± 7.18	0.018

PLT, platelets; Hb, Hemoglobin; APTT, activated partial thromboplastin time.

The secondary safety endpoint of bleeding events and gastrointestinal reaction occurred in 3 (15.79%) patients in the indobufen group and 14 (70%) patients in the aspirin group (*p* = 0.01). Total bleeding (BARC type ≥2) occurred in three patients (15.79%) in the indobufen group and 11 patients (55%) in the aspirin group (*p* = 0.011). There were 3 cases of gastrointestinal bleeding and a case of incision bleeding as major bleeding and 7 cases of hidden blood loss as minor bleeding in the aspirin group. The incidence of bleeding events, especially major bleeding events, was significantly lower in the indobufen group. Gastrointestinal complications occurred in three patients (15%) in the aspirin group and no patients in the indobufen group (*p* = 0.079), suggesting a safety advantage for the indobufen group.

Comparing bleeding and coagulation-related indicators and postoperative hospital stay, there was no significant difference in Hb, D-dimer levels and postoperative hospital stay between the indobufen group and the aspirin group (*p* > 0.05). However, PLT and APTT in the indobufen group were significantly lower than in the aspirin group (*p* = 0.001 and *p* = 0.018, respectively).

Considering that patients after CABG generally need to take dual antiplatelet drugs for 1 year and continue long-term oral antiplatelet drugs after stablization, the cost of antiplatelet drugs in both groups was calculated. Patients in the indobufen group received 100 mg bid daily, with the unit price of indobufen being 6.95 yuan/100 mg. The unit price of aspirin was 0.50 yuan/100 mg. Thus, the average daily cost of antiplatelet drugs in the indobufen group was 27.8 times higher than in the aspirin group. Long-term use of indobufen is expensive, and from a pharmacoeconomics perspective, indobufen is less cost-effective than aspirin.

## Discussion

Coronary heart disease is a leading cause of mortality with an increasing incidence, particularly among younger patients (Mahjoob et al., 2018). Platelet activation and aggregation are critical in atherosclerotic thrombosis development. While aspirin and clopidogrel combination therapy is common, some patients experience severe gastrointestinal reactions and poor tolerance to aspirin. Indobufen, a novel platelet aggregation inhibitor, offers minimal gastrointestinal effects (Yang et al., 2017; Chen et al., 2022; Wu et al., 2023; Lu and Xu, 2021).

The findings of this study provide valuable insights into the safety and efficacy of indobufen compared to aspirin in patients undergoing CABG. The results demonstrate a significant reduction in primary and secondary endpoint events, particularly bleeding complications, with indobufen, highlighting its potential as a safer alternative to aspirin in this patient population.

Our study revealed a notable difference in the occurrence of the primary composite endpoint between the indobufen and aspirin groups. The absence of primary endpoint events in the indobufen group compared to a 25% occurrence in the aspirin group underscores the effectiveness of indobufen in preventing adverse cardiovascular events post-CABG. This finding aligns with previous research suggesting that indobufen’s antiplatelet activity, while comparable to that of aspirin, may confer additional benefits in specific clinical scenarios due to its distinct pharmacological profile (Johnston et al., 2019).

The reduced incidence of non-fatal myocardial infarction, revascularization, stroke, and thrombotic events in the indobufen group further supports its efficacy. These outcomes are particularly relevant given the critical role of platelet activation and aggregation in the pathogenesis of atherothrombosis and subsequent cardiovascular events (Davì and Patrono, 2007; Gaudino et al., 2017). The results suggest that indobufen may offer a more robust protective effect against recurrent ischemic events in post-CABG patients.

One of the most significant findings of our study is the markedly lower incidence of bleeding events in the indobufen group. The secondary safety endpoint of bleeding events and gastrointestinal reactions was significantly lower in the indobufen group (15.79%) compared to the aspirin group (70%), with a *p*-value of 0.01. Major bleeding events, defined as BARC type ≥3, were notably fewer in the indobufen group. This finding is crucial because bleeding complications can significantly impact patient outcomes, leading to increased morbidity and mortality and necessitating additional medical interventions (CotpatCmd et al., 2018).

The lower incidence of gastrointestinal complications in the indobufen group, although not statistically significant (*p* = 0.079), indicates a trend toward better gastrointestinal tolerability compared to aspirin. This is an important consideration for long-term antiplatelet therapy, where gastrointestinal side effects are a common cause of medication discontinuation and non-compliance.

The study also assessed bleeding and coagulation-related indicators, such as Hb, D-dimer levels, and APTT. While there were no significant differences in Hb and D-dimer levels or postoperative hospital stay between the groups, PLT and APTT were significantly lower in the indobufen group. This finding suggests a more favorable coagulation profile with indobufen, potentially contributing to its lower bleeding risk.

Despite the clinical advantages of indobufen, the economic analysis highlights a significant drawback. The daily cost of indobufen therapy was substantially higher than that of aspirin, making long-term use less cost-effective. Given the necessity for prolonged antiplatelet therapy post-CABG, the high cost of indobufen could be a limiting factor for its widespread adoption, particularly in resource-limited settings. While indobufen demonstrated a superior safety profile compared to aspirin, its significantly higher cost—27.8 times more expensive—raises concerns about its cost-effectiveness for long-term use, especially in resource-limited settings. The economic burden of indobufen could restrict its accessibility, despite its clinical benefits. Future studies should include comprehensive pharmacoeconomic analyses to evaluate the cost-effectiveness of indobufen relative to other antiplatelet therapies.

### Limitation

Despite the promising findings, this study has several limitations. First, the small sample size and retrospective design limit the generalizability and introduce potential biases. Second the short follow-up period may not capture long-term outcomes, and the lack of blinding could lead to observer bias. Third, economic considerations were not thoroughly explored, and the homogeneity of the study population limits applicability to more diverse groups. Fourth, unmeasured confounding variables and the exclusion of certain patient groups restrict the findings. In addition, the loss of seven patients to follow-up (15.2% of the total study population) is another limitation that may have influenced our results. The reasons for loss to follow-up were not systematically recorded, which could introduce bias in the final outcomes. Future studies should implement strategies to minimize patient attrition and account for lost follow-up in their analyses. Future large-scale, multicenter, prospective studies with longer follow-up and comprehensive cost-effectiveness analyses are necessary to confirm these results and inform clinical practice.

## Conclusion

This study demonstrates that indobufen provides a comparable antiplatelet effect to aspirin, with a significant reduction in gastrointestinal reactions and bleeding events. Indobufen’s high selectivity and reversible receptor binding make it particularly suitable for aspirin-intolerant patients with high bleeding risk and gastric ulcers. Despite the promising results, this retrospective study’s limited sample size may introduce bias, and the shorter history of indobufen usage means its evidence base is less robust compared to aspirin.

Aspirin remains the preferred drug for primary and secondary prevention of cardiovascular and cerebrovascular diseases due to its established efficacy, availability, and cost-effectiveness. However, indobufen and other antiplatelet agents should be considered for patients who are intolerant to or contraindicated for aspirin. Further large-scale clinical trials are needed to validate these findings and to determine whether indobufen can replace aspirin as the first-choice antiplatelet therapy post-CABG.

## Data Availability

The original contributions presented in the study are included in the article/supplementary material, further inquiries can be directed to the corresponding authors.
